# Spin-polarized scanning tunneling microscopy with quantitative insights into magnetic probes

**DOI:** 10.1186/s40580-017-0102-5

**Published:** 2017-04-06

**Authors:** Soo-hyon Phark, Dirk Sander

**Affiliations:** 1 Center for Quantum Nanoscience, Institute for Basic Science, Seoul, 03760 Republic of Korea; 2 Department of Physics, Ewha Womans University, Seoul, 03760 Republic of Korea; 3 Max-Planck-Institut für Mikrostrukturphysik, 06120 Halle, Germany

**Keywords:** Spin-polarized scanning tunneling microscopy, Nanomagnetism, Magnetic anisotropy, Non-collinear magnetic order

## Abstract

Spin-polarized scanning tunneling microscopy and spectroscopy (spin-STM/S) have been successfully applied to magnetic characterizations of individual nanostructures. Spin-STM/S is often performed in magnetic fields of up to some Tesla, which may strongly influence the tip state. In spite of the pivotal role of the tip in spin-STM/S, the contribution of the tip to the differential conductance d*I*/d*V* signal in an external field has rarely been investigated in detail. In this review, an advanced analysis of spin-STM/S data measured on magnetic nanoislands, which relies on a quantitative magnetic characterization of tips, is discussed. Taking advantage of the uniaxial out-of-plane magnetic anisotropy of Co bilayer nanoisland on Cu(111), in-field spin-STM on this system has enabled a quantitative determination, and thereby, a categorization of the magnetic states of the tips. The resulting in-depth and conclusive analysis of magnetic characterization of the tip opens new venues for a clear-cut sub-nanometer scale spin ordering and spin-dependent electronic structure of the non-collinear magnetic state in bilayer high Fe nanoislands on Cu(111).

## Background

### Nanoscopic magnetic tunnel junction in STM

**Fig. 1:**
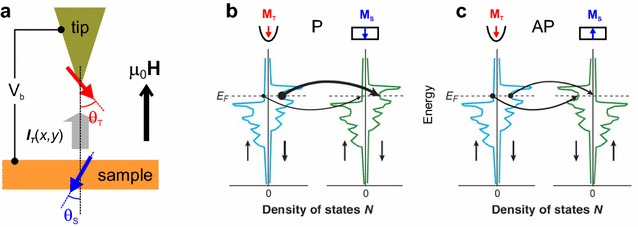
Tunneling in spin-polarized scanning tunneling microscopy. **a** A schematic illustration of the tunneling current between a magnetic sample and a magnetic tip in scanning tunneling microscopy (STM) in an external magnetic field μ_0_ *H*. **b**, **c** A simplified picture of spin-polarized tunneling within a hypothetical spin-split density of states model in parallel (*P*) and antiparallel (*AP*) magnetization orientations. The spin orientation of the tunneling electrons is assumed to be conserved during tunneling, i.e., spin-up electrons always tunnel into spin-up states and spin-down electrons always tunnel into spin-down states. Arrows (*bottom*) indicate the DOS of spin-up and spin-down electrons. The spin direction is antiparallel to the magnetic moment [64]. *M*_T_ and *M*_S_ (*top arrows*) denote the magnetization orientation of tip and sample, respectively. **b** *P* and **c** *AP* alignment of tip and sample magnetization (Reprinted from [4] with permission from American Physical Society (Copyright 2014))

Spin-polarized scanning tunneling microscopy and spectroscopy (spin-STM/S) is a magnetic imaging technique with an ultimate lateral resolution on the atomic scale [1–4], which is suitable for probing magnetism in ferromagnetic nanostructures [5–7] and in antiferromagnets [8–10]. Figure 1a schematically describes electron tunneling in spin-STM. Electrons carry spin as well as charge, thus the electron tunneling is spin dependent [11–13]. When magnetically ordered materials are used for both tip and sample, the tunneling current depends on the magnetic order parameters of both tip and sample (here the local magnetization, ***M***_T_ and ***M***_S_, respectively). In addition, spin-STM under an external field μ_0_ *H* allows to control the orientations of ***M***_T_ and ***M***_S_. This is the basis to use spin-STM, undoubtedly, to perform a ‘magnetic tunneling junction’ (MTJ) experiment on the single atom level.

A spatially resolved measurement of the differential conductance d*I*/d*V* of the tunnel current provides a spatial map of electronic information relevant to the local magnetic order. Figure 1b, c are schematics describing the spin-dependent tunneling in (b) parallel (*P*) and (c) antiparallel (*AP*) configurations between ***M***_T_ and ***M***_S_. The electronic density of states (DOS) of ferromagnets splits up into majority (*n* ^↑^)- and minority (*n* ^↓^)-spin DOSs due to exchange interaction between electrons [14], giving rise to the spin polarization *P*(*E*) at a given energy *E.*

1 $$P\left( E \right) = \frac{{n^{ \uparrow } (E) - n^{ \downarrow } (E)}}{{n^{ \uparrow } \left( E \right) + n^{ \downarrow } (E)}}$$

Assuming (a) identical DOSs for both sample and tip and (b) conservation of spin orientation during the tunneling process, the schematic implies that the tunneling current will depend on the magnetization configurations (*P* or *AP*), as depicted by the thickness of the curved black arrows. It is plausible that one can derive the contribution arising from the spin-polarization *P*(*E*) explicitly in the mathematical formula of the tunneling phenomena. Thereby, it is instructive to write the tunneling current at the tip position ***R***_T_ as

2 $$I\left( {\varvec{R}_{T} ;V;\theta } \right) = I_{0} \left( {\varvec{R}_{T} ;V} \right) + I_{P} \left( {\varvec{R}_{T} ;V;\theta } \right),$$

where *I*_0_ and *I*_P_ denote the non-spin-polarized and spin-polarized currents, respectively, and $\theta \;( \equiv \theta_{\text{T}} {-}\theta_{\text{S}} )$ is the angle between ***M***_T_ and ***M***_S_, as depicted in Fig. 1a.

### Spin-dependent differential conductance d*I*/d*V* in spin-STS

Wortmann et al. developed a theoretical description of the tunneling current in spin-STM [15]. According to Bardeen’s approach [16], the tunneling current is written as

3 $$I\left( {\varvec{R}_{{\mathbf{T}}} , V} \right) = \frac{2\pi e}{\hbar }\mathop \sum \limits_{\mu ,v} \left[ {f\left( {\upvarepsilon_{\mu }^{S} - \varepsilon_{F} } \right) - f\left( {\upvarepsilon_{\mu }^{T} - \varepsilon_{F} } \right)} \right] \times \delta \left( {\upvarepsilon_{\nu }^{T} -\upvarepsilon_{\mu }^{S} - eV} \right)\left| {M_{\nu ,\mu } \left( {\varvec{R}_{{\mathbf{T}}} } \right)} \right|^{2} ,$$

where ***R***_T_, *V*, $f(\upvarepsilon)$, $\upvarepsilon_{\mu /v}^{{{\text{T}}/{\text{S}}}}$, and $\upvarepsilon_{\text{F}}$ are the tip position, the bias voltage, the Fermi function, the tip/sample energy states, and the Fermi energy, respectively. To calculate the matrix elements $M_{\nu ,\mu } \left( {\varvec{R}_{\text{T}} } \right) = \langle{\varPsi }_{\nu }^{\text{T}} \left| {U_{\text{T}} } \right.\left| {{\varPsi }_{\mu }^{\text{S}} \rangle,} \right.$ the authors introduced the two component spinors for the tip and sample wave functions Ψ_*ν*_^T^ and Ψ_*μ*_^S^, respectively,

4 $$\varPsi_{\nu }^{T} = \left( {\begin{array}{*{20}c} {\psi_{\nu \uparrow }^{T} } \\ 0 \\ \end{array} } \right)\quad {\text{or}} \quad \varPsi_{\nu }^{T} = \left( {\begin{array}{*{20}c} 0 \\ {\psi_{\nu \downarrow }^{T} } \\ \end{array} } \right) ,\quad \varPsi_{\mu }^{S} = \left( {\begin{array}{*{20}c} {\psi_{\mu \uparrow }^{S} } \\ {\psi_{\mu \downarrow }^{S} } \\ \end{array} } \right)$$

The authors also introduced the ‘magnetization density of states’ ***m***(*E*) to account for the decisive role of the relative orientation between ***M***_T_ and ***M***_S_ in the tunneling of spin-STM,

5 $$\varvec{m}\left( E \right) = \left( {n^{ \uparrow } - n^{ \downarrow } } \right)\hat{\varvec{e}}_{\text{M}} = nP\left( E \right)\hat{\varvec{e}}_{\text{M}} ,$$

where *n* ^↑^ (*n* ^↓^) is the spin-up (-down) DOS at energy *E*, and $\hat{\varvec{e}}_{\text{M}}$ is the unit vector of the magnetization. Assuming spin-conserved tunneling and constant tip DOS [17], substitution of the spinors (Eq. 5) leads to

6 $$\begin{aligned} I\left( {\varvec{R}_{\text{T}} , V,\theta } \right) = \frac{{8\pi^{3} C^{2} \hbar^{3} e}}{{\kappa^{2} m^{2} }}\mathop \int \nolimits {d\varepsilon } \left[ {f\left( {\upvarepsilon -\upvarepsilon_{F} } \right) - f\left( {\upvarepsilon + eV -\upvarepsilon_{F} } \right)} \right]\mathop \sum \limits_{\mu } \delta \left( {\upvarepsilon_{F} -\upvarepsilon} \right) \hfill \\ \;\;\;\;\;\;\;\;\;\;\;\;\;\;\;\;\;\;\; \times \left[ {n_{\text{T}}^{ \uparrow } \left| {\psi_{\mu \uparrow }^{\text{S}} \left( {\varvec{R}_{\text{T}} } \right)} \right|^{2} + n_{\text{T}}^{ \downarrow } \left| {\psi_{\mu \downarrow }^{\text{S}} \left( {\varvec{R}_{\text{T}} } \right)} \right|^{2} } \right], \hfill \\ \end{aligned}$$

where $\theta \left( {\varvec{R}_{\text{T}} , V} \right)$ denotes the angle between ***M***_T_ and ***M***_S_.

The differential conductance d*I*/d*V* measurement in the spin-STS provides a quantity which is directly correlated to the projection of the spin-DOS of the sample onto that of the tip at the tip position ***R***_T_. Recalling the description of Eq. 2, and using a set of algebraic processes [15], the Eq. 6 gives

7 $$\frac{{\text{d}I}}{{\text{d}V}}\left( {\varvec{R}_{\text{T}} , V} \right) \propto n_{\text{T}} n_{\text{S}} \left( {\varvec{R}_{\text{T}} ,\upvarepsilon_{\text{F}} + eV} \right) + \varvec{m}_{\text{T}} \cdot \varvec{m}_{\text{S}} \left( {\varvec{R}_{\text{T}} ,\upvarepsilon_{\text{F}} + eV} \right),$$

which exhibits a non-magnetic (1st term; d*I*/d*V*|_0_) and magnetic (2nd term; d*I*/d*V*|_mag_) term explicitly. Note, that the second (magnetic) term results in $n_{\text{T}} n_{\text{S}} P_{\text{T}} P_{\text{S}} \hat{\varvec{e}}_{\text{M}}^{\text{T}} \cdot \hat{\varvec{e}}_{\text{M}}^{\text{S}}$, which is proportional to the projection of ***M***_S_ to ***M***_T_ at the tip position.

### Experimental approach

The experiments were performed in situ in an ultra-high vacuum (UHV) chamber (base pressure <1 × 10^−11^ mbar) equipped with a liquid He cooled scanning tunneling microscope operating at 10–30 K and a superconducting magnet for field of up to 7 T normal to the sample surface [4, 18].

#### Co and Fe nanostructures on Cu(111)

**Fig. 2:**
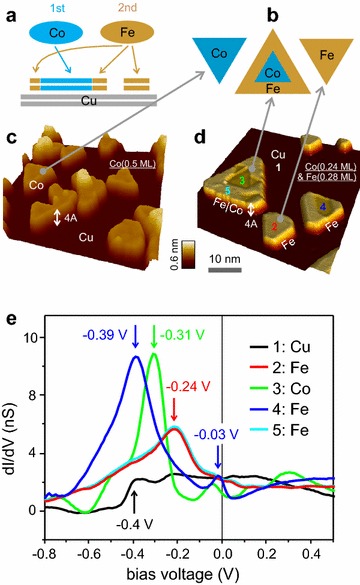
Preparation of biatomic-layer-high Co and Fe nanoislands on Cu(111). **a** An illustration showing the sample preparation. A submonolayer (sub-ML) of Co was deposited first on the Cu(111) substrate. Then a sub-ML of Fe was deposited subsequently. **b** CC-STM image of the sample surface after Co deposition of an amount equivalent to 0.5 ML. (50 × 50 nm^2^; *V*_b_ = 0.1 V; *I*_set_ = 2 nA). **c** STM image of the sample surface after 0.24 ML Co deposition followed by subsequent 0.28 ML Fe deposition. (50 × 50 nm^2^; *V*_b_ = –0.3 V; *I*_set_ = 3 nA). **d** A cartoon showing the lateral structures of 3 types of the nanoislands obtained by the sample preparation process illustrated in a. e, Differential conductance d*I*/d*V* curves measured by scanning tunneling spectroscopy (STS) at the positions marked 1–5 in **c**. *Position mark* in **c** and corresponding d*I*/d*V* curve in **d** of each STS measurement share the same color code

Here we focus on biatomic-layer-high (BLH) Co, Fe, and Fe-decorated Co (Fe|Co) islands on Cu(111) [7, 18–20]. Figure 2a schematically illustrates the sample preparation procedure. It results in 3 types of BLH islands schematically depicted in Fig. 2b: First, a sub-monolayer (ML)-equivalent Co deposition at room temperature (RT) forms Co islands as shown in Fig. 2c. Constant current STM (CC-STM) image of the sample surface formed by 0.5 ML-equivalent Co reveals an island height of ~4 Å, indicative of BLH Co. The islands have a lateral dimension ranging from 5 to 30 nm. Second, the sequential deposition of first sub-MLs of Co (0.24 ML) and then Fe (0.28 ML) at RT formed two types of islands (Fig. 2d): (1) pure BLH Fe island; (2) BLH Fe|Co island, where Co forms the core and Fe surrounds the Co perimeter. We performed spin-polarized STM and STS on individual islands for all three types of islands.

#### Spectroscopic identification of element-specific electronic structures of nanoislands

We discriminate different surface regions (Co, Fe, and Cu) in the STM images (Fig. 2c and d) by differences in apparent heights. Those regions are also identified spectroscopically by local STS measurements. Figure 2e shows five STS spectra measured at the positions indicated by the numbers 1–5 in Fig. 2d, where the same color code is used. Spectra 1 and 3 exhibit the onsets of the surface states of Cu(111) [21] and the 3*d*_z2_-minority state of Co [22] at the respective characteristic bias voltages *V*_b_ = −0.4 and −0.3 V. STS identifies two electronically different Fe regions. BLH Fe on Cu(111) has two structurally and electronically different phases [23, 24]. Spectrum 4 shows a dominant peak at −0.39 V and a small peak at −0.03 V. This is the signature of the electronic structure of BLH Fe in fcc-stacking [24]. Spectrum 5 shows a peak at −0.22 V, and it is almost indistinguishable from the spectrum measured at position 2 in the pure Fe island. These spectra indicate BLH Fe in bridge-site-stacking of topmost Fe atoms [23, 24].

#### Spin-polarized STM/S and d*I*/d*V* mapping

For non-magnetic STM/S measurements, we used electrochemically etched W tips. For spin-STM/S, we used either Fe-coated W tips [19] or Cr/Co-coated W tips [5, 7, 25]. Details of the sample and tip preparations are described in Refs. [7] and [19]. STS spectra were measured by employing a lock-in technique with a modulation *V*_b_ at a frequency *v* = 4 kHz and a root-mean-square amplitude of 20 mV to detect the tunnel current *I*(*V*) and the differential conductance d*I*/d*V* simultaneously. In order to obtain the switching field *H*_sw_ of an individual island, the differential conductance magnetic hysteresis loop d*I*/d*V*(*H*) was measured at the center of the Co core of the island with an external field μ_0_ *H* sweep up to 3 T. The *H*_sw_ of the island was extracted from the sharp drop of the signal in the d*I*/d*V*(*H*) hysteresis loop. For a simultaneous measurement of CC-STM and spin-polarized d*I*/d*V* images, we utilized the spectroscopic mapping technique under an external magnetic field. The field value and the field history is chosen such that at the same field value *AP* and *P* magnetization configurations are realized. After taking STS spectrum at each location of a scan for varying the bias voltage, we extract the d*I*/d*V* signal for every pixel at a given energy. Thus, we obtain energy-resolved d*I*/d*V* maps of an island for both *AP* and *P* magnetization configurations. To obtain a spatial resolution on the atomic scale (<2 Å), we chose a pixel number larger than 150 × 150 for a 25 × 25 nm^2^ image size. This corresponds to a measurement time of 12 h order for a spectroscopic map.

## Field dependence of differential conductance in spin-stm

### Spin-STM of Co nanoislands on Cu(111)

**Fig. 3:**
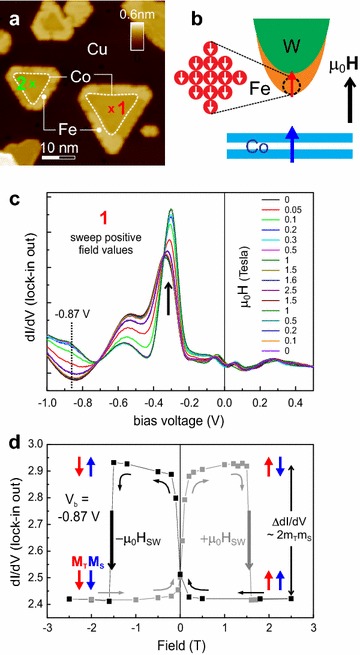
Magnetic hysteresis of d*I*/d*V* in spin-polarized STM/S. **a** STM image of two triangular Co cores of Fe-decorated Co islands on which the magnetic-field-dependent d*I*/d*V* were measured. The inset shows the apparent STM height profile along the white solid line. (*V*_b_ = −0.2 V; *I*_set_ = 1 nA). **b** A schematic of spin-STM measurement on a bilayer Co island in an external magnetic field μ_0_ *H*. **c** A set of d*I*/d*V* curves measured under a varying μ_0_ *H*_ext_ at the position “1” marked in a. (*V*_stab_ = 0.5 V; *I*_stab_ = 1 nA). **d** Magnetic hysteresis extracted from the field dependence of d*I*/d*V* values at *V*_b_ = −0.85 V (**c**) (Reprinted from [19] with permission from American Institute of Physics (Copyright 2013))

Figure 3a shows a STM image of typical BLH Fe|Co islands on Cu(111). The Co core regions of the islands are enclosed by dotted lines and labeled by ‘1’ and ‘2’, [19]. The crosses mark the location of the d*I*/d*V* measurements. Spin-STM measurements were carried out using Fe-coated W tips in an external magnetic field μ_0_ *H*, as schematically illustrated in Fig. 3b, at a sample temperature of 10 K.

Figure 3c shows the STS spectra measured at the center of Co core 1, as marked by the red cross in Fig. 3a, under varying μ_0_ *H* along the surface normal. The STS spectra show at all field values the sharp 3*d*_z2_-related electronic state of Co near *V*_b_ = −0.3 V [22], as indicated by the black vertical arrow. Shape and amplitude of the spectra change with magnetic field. We identify a bias voltage *V*_b_ = −0.87 V, which gives a clear field-dependent change of the d*I*/d*V* signal. Figure 3d shows the d*I*/d*V* hysteresis loop extracted from the d*I*/d*V* values at *V*_b_ = −0.87 V. The hysteresis loop consists of butterfly-shaped curves, which are symmetric about the vertical axis [25, 26]. The sudden drop of the signal at ±1.6 T indicates the switching of the magnetization direction of the Co core [6]. A gradual change of the d*I*/d*V* signal is observed in the field range below the switching field *H*_sw_, implying that the out-of-plane component of the tip magnetization varies with the external field [25].

### Probing methods of spin-STM

#### Spin-polarized tips

Spin-polarized tips are an important ingredient for spin-STM. Several experimental schemes for obtaining spin-polarized tips have been proposed, which were already realized in planar tunnel junctions: (1) magnetic materials [13, 27–29], (2) optically pumped GaAs [30], and (3) superconducting materials in magnetic fields [11, 12, 31, 32] have been used in spin-dependent transport measurements.

#### Modulation of tip magnetization method

A mode of spin-STM operation, where a tiny coil is used for periodically switching the tip magnetization back and forth, was introduced by Wulfhekel et al. [33]. If the modulation frequency exceeds the cutoff frequency of the feedback loop, the measured signal becomes proportional to the local magnetization of the sample. This method can effectively separate topographic and electronic from magnetic contrast effects for a given sample bias. However, this approach appears to be highly demanding and time consuming compared to the simple measure of the STM signal using a spin-polarized tip. Another limitation of this method is that ferromagnetic tips have to be used, which is not free from the effect of its stray field on the measured magnetic contrast. Despite, recently near-atomic resolution has been achieved on a reconstructed Mn film epitaxially grown on a Fe(001) substrate using the modulated tip magnetization mode [34].

#### Tunneling anisotropic magnetoresistance (TAMR) effect

There are some reports which claim to observe spin-contrast for tips with no spin-polarization [35–37]. In principle the tunnel current—even from a non-spin-polarized tip—depends on the magnetization direction of the sample and a magnetic contrast is feasible. This tunneling anisotropic magnetoresistance (TAMR) has been reported for W-tips. The significance of TAMR for spin-STM is difficult to judge, as material exchange form the magnetic sample onto the tip cannot be excluded exclusively.

### Tips made of magnetic materials

All results presented in this review were obtained using tips of magnetic materials. Common ways of preparing tips of magnetic materials are either coating W-tips with magnetic materials or direct electrochemical etching of bulk magnetic materials [38, 39]. Using a bulk antiferromagnetic material such as Cr [25, 26] or MnNi diminishes the magnetic stray field emanating from the tip [40–43].

#### W tips coated by magnetic materials

A few sequential steps are applied to obtain Fe [19, 20], Cr [25], Cr/Co [5, 7, 25] -coated W tips: (1) ex situ preparation of W tips through electrochemical etching in 1.5 M NaOH solution. (2) flash heating of the etched W tip at a temperature above 2200 °C under UHV conditions to remove contaminants and W-oxides, and to partially crystallize the tip apex. (3) deposition of magnetic materials, such as Fe (40 AL-equivalent), Cr (20–100 AL-equivalent), or Co (40 AL-equivalent) followed by Cr (40 AL-equivalent) onto W tip apices for Fe-W, Cr-W, or Cr/Co-W tips, respectively. This deposition is followed by post-annealing at a mild temperature around 400 °C to recrystallize each magnetic coating for a stable structural phase at the tip apex. (4) in situ microscopic preparation by application of voltage pulses between tip and sample with a duration of typically a few ms to shape the tip apex. Just after each voltage pulse, the tip was tested by STS measurement on a surface with well-known spectroscopic features, such as the surface states of Cu(111) and bilayer Co island. The proper d*I*/d*V* signal indicates that the tip is usable in spin-STM measurements.

#### Cr-bulk tips

The tip was made of polycrystalline Cr rods with a nearly square cross section of 0.7 × 0.7 mm^2^ obtained by cutting a 99.99% Cr foil (from Super Conductor Materials). The Cr rod was electrochemically etched in 1.5 M NaOH solution, following the procedure reported in Ref. [38] and then it was directly transferred into the STM. The only in situ preparation step was the application of voltage pulses on the Cu(111) surface to shape the apex [26].

#### Characterization of tips by field emission microscopy (FEM)

In the field emission microscopy (FEM), the electrons are extracted from the metal tip to the vacuum [44], thus, their spin polarization is proportional to that of the surface at the metal tip. Nagai et al. investigated the atomic structures and spin polarizations at the apex of Fe/W and Cr/W tips used in spin-STM by means of FEM and field ion microscopy (FIM) [45]. FIM has been used to investigate atomic structures at the apex of metal tips [46]. Thus, FEM combined with FIM can provide information of spin polarization of a tip correlated to its atomic structure. The FIM of the Fe/W tip showed that the deposited Fe layers form a single crystalline structure over the apex, except for the first few layers. The spin polarization at the surface is as high as 41.2%, which is attributed to the single crystalline structure of Fe. On the other hand, a FIM image of the Cr/W tip showed that the Cr layer was not well-ordered crystalline, although some hints of crystalline order were observed because of the Cr islands. Consequently, the spin polarization is as low as 10.3%. These results may allow one to prepare and characterize the structure of the magnetic tips and to quantify their spin polarization for use in spin-STM.

### Magnetic responses of tips to the external field

**Fig. 4:**
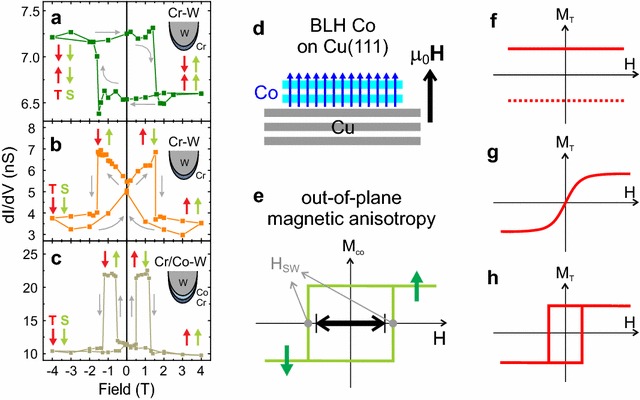
Magnetic response of tips in spin-STM. **a**–**c** d*I*/d*V* magnetic hysteresis loops measured on bilayer Co islands, with 3 different tips as denoted at the *top*-*left* corner of each figure. The *black arrows* show the field sweep directions for data acquisition. The *red* and *green arrows* represent the magnetization directions of tip and sample, respectively, at field values for d*I*/d*V* signal saturation. For the loop in a, a fixed tip magnetization direction is expected, however, its direction cannot be decided and the two possibilities are shown. **d**, **e** Schematics for local magnetic moments (**d**) and magnetization hysteretic response (**e**) of a bilayer Co island on Cu(111). **f**–**h** Field response of the tip magnetization extracted from the d*I*/d*V* hysteresis loops shown in **a**–**c**, respectively, in regard to the magnetic response of the Co island described in **e** (Reprinted from [25] with permission from American Institute of Physics (Copyright 2009))

Figure 4a–c show magnetic hysteresis loops of the d*I*/d*V* signal measured at the center of bilayer Co islands on Cu(111) using (a and b) Cr-W and (c) Cr/Co-W tips [25]. A first inspection of the data reveals a striking unexpected result that the same macroscopic preparation procedure of tips gives rise to vastly different hysteresis curves (compare Fig. 4a, b). To appreciate the shape of the hysteresis curves in Fig. 4a–c, it is important to realize that the field-driven change of the d*I*/d*V* signal is ascribed to a corresponding change of the relative orientation between the ***M***_T_ and ***M***_S_ [1].

The observed hysteresis curves are either (a) asymmetric or (b and c) symmetric about the vertical d*I*/d*V* axis. Figure 4d, e are schematic illustrations of the local magnetization configurations and of the magnetic response of a bilayer Co island to the external field, respectively. Due to the uniaxial out-of-plane magnetic anisotropy, the Co island exhibits only a bistable single domain magnetization state. The Wortmann formula (Eq. 7) consequently points at the response of the tip to the applied field, as schematically illustrated in Fig. 4f–h, respectively. Accordingly, the shape of the curve in Fig. 4a is ascribed to a fixed magnetization direction of the tip apex, which does not change in response to the external field, as sketched in Fig. 4f. On the other hand, the symmetric hysteresis curves in Fig. 4b and c indicate that here the magnetization direction of the tip apex has changed in response to the external field. The magnetic response in Fig. 4g indicates a gradual increase with external field of the out-of-plane component of the tip magnetization *M*_T,⏊_ along the field direction, starting from 0 at zero field, and reaching saturation before the BLH Co core switches. A non-hysteretic field dependence of d*I*/d*V*(*H*) results for *H* < *H*_sw_. Figure 4h represents a ferromagnetic state of the tip, where a switching of tip and sample at different field magnitudes is observed. The results presented in Fig. 4 imply a complex magnetic behavior of the tips, which even varies under the same macroscopic preparation. This indicates that, in addition to the macroscopic preparation, also the in situ microscopic tip preparation by voltage pulses is a further decisive aspect which determines the magnetic contrast in spin-STM.

Note that only the tips characterized by curves in Fig. 4a, c give rise to a magnetic contrast for an out-of-plane magnetized sample at zero field. Tips represented by the curve in Fig. 4b require an out-of-plane field to provide magnetic contrast for out-of-plane magnetized samples. Therefore, the results of Fig. 4 indicate that the magnetic contrast in spin-STM changes differently for a change of magnetic configuration of the tip.

It has been tacitly assumed that a high signal of the d*I*/d*V* in spin-STM corresponds to a *P* configuration, while a low signal corresponds to an *AP* configuration [47–49]. However, the results presented in Fig. 4 show that only in connection with a field sweep, a reliable deduction of the magnetic configurations from the d*I*/d*V* signal is warranted. A closer inspection of Fig. 4 reveals that the value of the d*I*/d*V* and its variation upon a change of the magnetic configuration varies from curve to curve, and it also differs for the same macroscopic preparation. The considerable difference of the magnetically induced signal Δd*I*/d*V* (refer Fig. 3d; ~0.6 nS for Fig. 4a, ~3 nS for Fig. 4b) also reflects that a specific magnetic behavior is not necessarily linked to a certain macroscopic tip preparation. All the observations in Fig. 4 indicate that neither the *material* at the tip apex, nor its *thickness*, nor its *spectroscopic features* are parameters which are sufficient to determine the magnetic properties of the tip, but only field-dependent measurements do so.

## Magnetic characterization of tips in spin-stm

To understand the tip contribution to the a d*I*/d*V*(*H*) signal requires a plausible physical description of ***M***_T_. We introduced in Sect. 2.4 various types of magnetic response of ***M***_T_ to the applied field. However, it is still not sufficient due to the following complications: (1) The non-hysteretic field response illustrated in Fig. 4g can be caused by either a field-driven continuous rotation of ***M***_T_, which is originally in-plane oriented at zero-field, or by a superparamagnetic (SPM) response of ***M***_T_. In both cases the out-of-plane component of the tip magnetization *M*_T,⏊_ increases with field, and a non-hysteretic and saturated field dependence of d*I*/d*V*(*H*) results. (2) The tip can also have an out-of-plane magnetic anisotropy with a large magnetic moment and exhibits a ferromagnetic response to the applied field, as illustrated in Fig. 4h. This reasoning can be applied to all cases shown in Fig. 4. This implies that the sharp change in all three d*I*/d*V*(*H*) curves could alternatively stem from the switching of the bistable ***M***_T_ along the applied field direction instead of that of ***M***_Co_. In this section, we introduce some experimental approaches of resolving the abovementioned open issue and, thus, advancing spin-STM to be a tool for the quantitative measurement of nanomagnetism in samples by specific control of the external field.

### Ferromagnetic tip

**Fig. 5:**
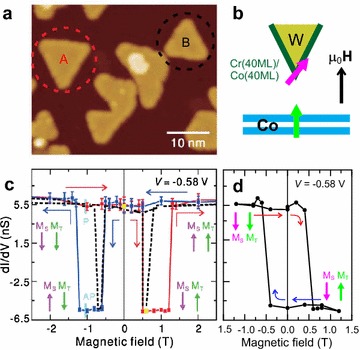
d*I/*d*V* hysteresis loops of Co islands measured with a ferromagnetic tip. **a** CC-STM image of Co islands on Cu(111) (*V*_b_ = −0.1 V, *I*_set_ = 1 nA). **b** Schematic illustration for the spin-STM measurements shown in **c** and **d**. Note the tip material configuration. **c**, d*I/*d*V* hysteresis loops at the center of the Co islands A and B marked by the *red* and *black dashed circles*, respectively, in **a**. The *red* (*blue*) *color* is for the measurements on the island A and corresponds to forward (*backward*) sweep of the magnetic field. The *black dashed line* presents a d*I/*d*V* hysteresis loop of the island B in **a**. **d**, Minor hysteresis loop taken at the center of island A, with the sample magnetization ***M***_S_ pointing down as indicated (Reprinted from [4] with permission from American Physical Society (Copyright 2014))

Figure 5a is a CC-STM image of bilayer Co islands on Cu(111) [50, 51]. Spin-STM of the Co islands was performed with a Cr/Co-coated W tip, as schematically described in Fig. 5b. Differential conductance spectra were measured at the center of two Co islands, marked by the dashed circles (red and black) in Fig. 5a. A pronounced magnetic field dependence of the d*I*/d*V* signal is observed [5], in the d*I*/d*V* hysteresis loops. Note the sharp signal drop at μ_0_ *H* = ±0.5 T is observed on both islands. The steep jumps at μ_0_ *H* = ±1.3 and ±0.8 T differs for red and black island. The switching field *H*_sw_ of the magnetization reversal of the BLH Co islands on Cu(111) strongly depends on the size of the islands [6, 47]. On the other hand, the *H*_sw_ of the tip would be the same provided the d*I*/d*V* measurements are performed with the tip apex. Thus, the sharp signal drop at μ_0_ *H* = ±0.5 T must be ascribed to the reversal of ***M***_T_, and the steep increase at μ_0_ *H* = ±1.3 and ±0.8 T is ascribed to the reversal of the magnetization of the larger (black) and the smaller (red) Co islands, respectively. The minor hysteresis loop in Fig. 5d, obtained under a field sweep between −1.2 and +1.2 T, where ***M***_S_ keeps its direction pointing down, corroborates the discussion on the response of ***M***_T_ to the external field. Here, a ferromagnetic tip with a switching field of ±0.5 T is observed.

### Superparamagnetic tips

Magnetism on a nanometer scale strongly depends on the measurement temperature due to the impact of thermal fluctuations. This is described by the so called ‘superparamagnetic criterion’ 25*k*_B_ *T* ≥ *K*_a_ *V*, where *k*_B_ *T* and *K*_a_ *V* represent the thermal energy and the magnetic anisotropy energy, respectively [52]. If the tip response shown in Fig. 4g is caused by the field-driven rotation of the ***M***_T_ from in-plane to out-of-plane, the magnetic anisotropy energy should be large enough to maintain the given magnetic state against thermal fluctuations. On the other hand, the field dependence of the d*I*/d*V* signal will depend strongly on the measurement temperature in case of a tip in the superparamagnetic state.

**Fig. 6:**
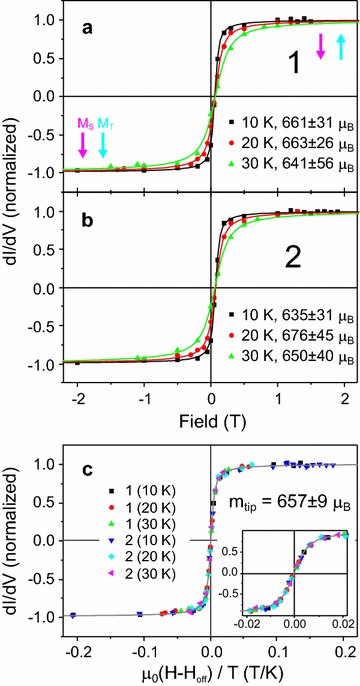
Superparamagnetic tip. **a**, **b** Magnetic-field-dependent d*I*/d*V* plots in **a** and **b** extracted from the hysteresis loops of Fig. 2c, d respectively. The d*I*/d*V* curves at each temperature are normalized by fits using Eq. 8, as shown by the *solid curves*. The legend gives the measurement temperature and the total magnetic moment in μ_B_, as extracted from the fit of the data with the Eq. 8. **c**, Plots of all d*I*/d*V* values as a function of reduced magnetic field (μ_0_ *H*/*T*). The *inset* is a close-up of the plots around μ_0_(*H*–*H*_off_)/*T* = 0. The *gray curve* shows the Langevin fit in one curve for all data (Reprinted from [19] with permission from American Institute of Physics (Copyright 2013))

To address this ambiguity and to elucidate the physical origin of the tip response shown in the butterfly-shaped d*I*/d*V*(*H*) in Fig. 4b, we measured d*I*/d*V*(*H*) hysteresis curves at different temperature, where ***M***_Co_ retains a fixed orientation (here pointing down). Figure 6a, b present the field dependence of d*I*/d*V* data measured at the center of the Co cores of the islands 1 and 2 shown in Fig. 3, respectively. We used a Fe-W tip at three temperatures, 10 K (black), 20 K (red), and 30 K (green). Note that the slope of the d*I*/d*V* signal at 0 T gets smaller with increasing temperature. At a fixed orientation of ***M***_Co_, we ascribe the observed change of the d*I*/d*V*(*H*) signal to the response of the tip to both field and temperature. Each data set of the same color code shows a saturation with increasing field, but the saturation field increases with increasing temperature. This suggests a superparamagnetic response of the tip.

To support and quantify this assertion, we analyze the temperature dependences of the d*I*/d*V* signal in Fig. 6a and b within the framework of superparamagnetism [52] by fitting each set of d*I*/d*V* data to a Langevin function

8 $$\frac{dI}{dV}\left( H \right) = \left( {\frac{dI}{dV}} \right)_{\text{sat}} \left[ {\coth \frac{{m_{\text{tip}} \mu_{0} \left( {H - H_{\text{off}} } \right)}}{{k_{\text{B}} T}} - \frac{{k_{\text{B}} T}}{{m_{\text{tip}} \mu_{0} \left( {H - H_{\text{off}} } \right)}}} \right] - \left( {\frac{dI}{dV}} \right)_{\text{C}}.$$

We introduce the saturation of the differential conductance (d*I*/d*V*)_sat_ and the differential conductance offset (d*I*/d*V*)_C_, which determines the average of the two d*I*/d*V* saturation values for the *P* and *AP* magnetization configurations. We also introduce the offset field *H*_off_, to consider the shift of the curves by 60 mT to the positive field direction, which tentatively ascribed to the stray field induced by ***M***_Co_. The d*I*/d*V* data are normalized to a saturation value of ±1. The solid curves through the data points of Fig. 6a, b are fits using Eq. 8, resulting in a magnetic moment *m*_tip_ = 660 ± 30 μ_B_ for all measurements.

The convincing description of the experimental data by the Langevin approach is further corroborated by plotting all data points as a function of μ_0_ *H*/*T*. The condensation of all data on a single curve, as shown in Fig. 6c, is the hallmark of a superparamagnetic response [52]. In a first approximation, we ascribe the extracted magnetic moment to a macrospin, where all individual spins at the tip apex respond in unison to the external field. Assuming a magnetic moment of 2.2–3 μ_B_ per single Fe atom in Fe nanoclusters [53] leads us to speculate that a nano-apex of approximately 220–300 Fe atoms determines the magnetic response of this tip.

We use the ‘superparamagnetic criterion’ [52], to estimate the upper limit for the magnetic anisotropy as *K*_a_ ≤ 0.07–0.1 meV/atom for 300–220 Fe atoms, respectively. Thus, even a considerably increased magnetic anisotropy per Fe atom of the nano-apex as compared to bulk Fe (~0.0035 meV/atom) [54] would still fulfill the superparamagnetic criterion. In spite of the convincing description of our data with a macrospin model, we cannot exclude a more complicated arrangement of the magnetic structure at the nano-apex, which could also result in the same magnetic moment of 660 μ_B_. We analyzed seven Fe-coated W tips, which were prepared under the same conditions as described above. In all cases we found a very good description of the experimental data by a superparamagnetic response. The analysis revealed a magnetic moment between 100 and 2000 μ_B_ for the different tips. This finding suggests that the same macroscopic tip preparation by Fe deposition followed by annealing may lead to different nano-apices with 30–900 Fe atoms, and these microstructures at the tip apex determine the magnetic response of the tip.

### Tips with a Stoner-Wohlfarth-like magnetization response

**Fig. 7:**
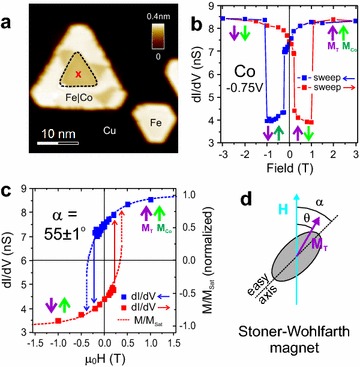
Stoner-Wohlfarth response of spin-STM tip. **a** A STM image showing a Fe|Co island and Fe island (*V*_b_ = −0.3 V, *I*_set_ = 3 nA). The black dashed curve encloses the Co core. **b** and **c** Magnetic hysteresis loops of d*I*/d*V* signals (*V*_b_ = −0.75 V) of the STS spectra measured at the center (marked by the *cross*) of the Co core of the island A (field sweep between −3 T and +3 T for **b**; field sweep between −1 T and +1 T for **c**). *Red* (*blue*) *color* code denotes the d*I*/d*V* values measured for the forward (*backward*) field sweep. The *purple* and *green arrows* indicate the directions of the magnetizations of the tip and Co core, respectively. Note that the Co magnetization ***M***_Co_ retains its orientation in **c**. The *dashed curves* in **c** are fits according to the SW model of the data with as a fit parameter. The *solid* (*dashed*) *red arrows* indicate the magnetization switching of the tip (the SW-magnet). **d** Description of the tip magnetization vector ***M***_T_ based on a SW model. *α* and *θ* represent the polar angles of the magnetic easy axis and the magnetization vector, respectively, with respect to the direction of the external magnetic field (along the *z*-axis) (Reprinted from [7] with permission from Nature Publishing Group (Copyright 2014))

Figure 7a shows a CC-STM image of a BLH Co island in contact with a Fe bilayer rim (Fe|Co island) [18, 19] and a pure Fe island. We performed field-dependent d*I*/d*V* measurements at the center of the Co core, which is indicated by the black dashed curve of the island A. We used a Cr/Co-coated W tip under an external field sweep between −3 and +3 T [7]. Figure 7b shows the d*I*/d*V* hysteresis loop, extracted from the d*I*/d*V* values of the Co spectra. It clearly shows two distinctive groups of d*I*/d*V* values, corresponding to the *AP* (intermediate field values) and *P* (high field values) magnetization configurations [5]. The changes of the d*I*/d*V* signal at ±0.24 T and ±1.1 T indicate the magnetization reversals of the tip and of the Co core, respectively, as discussed in the Sect. 3.1. Interestingly, a close inspection of the curve after subtraction of the signal change due to sample and tip reversal reveals a monotonic change of the d*I*/d*V* signal, which saturates at ∼ ±1.5 T.

To identify the magnetic state of the tip, we obtained a d*I*/d*V* hysteresis loop with a field sweep between −1 and +1 T, as shown by red and blue squares in Fig. 7c. This field regime is smaller than the switching field of the Co core, thus leaving the ***M***_Co_ unchanged. Therefore, the field dependence of the d*I*/d*V* signal in Fig. 7c reflects solely the response of ***M***_T_ to the applied field. Besides the magnetization switching at ±0.24 T, we note a monotonic d*I*/d*V* signal increase (decrease) for the backward (forward) field sweep. Based on the constant ***M***_Co_ oriented in the out-of-plane direction, the field-dependence of the d*I*/d*V* signals in Fig. 7c implies a gradual increase of the out-of-plane component of the ***M***_T_ for an increasing external field.

To obtain a quantitative insight into the tip magnetization ***M***_T_, we apply a Stoner-Wohlfarth (SW) model [55] (Fig. 7d) to the d*I*/d*V* hysteresis in Fig. 7c. The SW-model employs two angles *α* and *θ*, which represent the tilting of the magnetic easy axis and of the magnetization vector, respectively, from the axis of the external field. The field-dependence of the magnetization in the SW-model is described by the equation

9 $$h\left( m \right) = - \frac{{Am\sqrt {1 - m^{2} } + B\left( {1 - 2m^{2} } \right)}}{{\sqrt {1 - m^{2} } }},$$

where *A* = *cos* ^2^α − *sin* ^2^α and *B* = *cos*α·*sin*α. Here the reduced magnetization *m* ≡ *M*/*M*_sat_ = *cos*θ and the reduced external magnetic field *h* ≡ *μ*_0_ *M*_sat_ *VH*/2*K*_a_, where *M*_sat_, *V*, and *K*_a_ represent the saturation magnetization, particle volume, and magnetic anisotropy constant, respectively, are used. The fit of the d*I*/d*V* hysteresis in Fig. 7c to Eq. 9 (dotted curves with dotted arrows) results in *α* = 55 ± 1°. This implies that ***M***_T_ is tilted by 55 ± 1° at zero-field, away from the sample normal, as shown schematically in Fig. 7d.

## Spin-stm with quantitatively characterized magnetic tips

In this section, we introduce some recent applications of spin-STM to unravel non-collinear spin ordering and spin-dependent electronic structures in nanometer scale magnetic structures, based on the quantitative characterizations of tips as discussed in the last section.

### Non-collinear spin order probed by field-tuned tips

#### Experimental proof of noncollineartiy of a periodic spin-dependent d*I*/d*V* pattern

A spatially periodic magnetic state could originate from either a collinear [56, 57] or non-collinear [9] spin-density wave (SDW). A collinear-SDW is characterized by a periodic change of the magnitude of the spin moments, while the spin orientation is fixed. On the other hand, a non-collinear SDW is composed of spin moments of constant magnitude but with changing orientation. The d*I*/d*V* signal in spin-STS depends on $\hat{\varvec{e}}_{\text{T}} \cdot \hat{\varvec{e}}_{\text{S}}$ (Eq. 7) [15]. If one uses a tip of in-plane oriented ***M***_T_ at zero field, as induced by an in-plane magnetic anisotropy, the tip is sensitive only to the in-plane component of ***M***_S_ (*M*_S,||_) at zero field. On the other hand, the tip will be sensitive only to the out-of-plane component of ***M***_S_ ($M_{{{\text{S}},{ \bot }}}$) at a field large enough to saturate ***M***_T_ along the field direction. Accordingly, magnetic-field-dependent d*I*/d*V* measurement for a collinear-SDW will result only in a change of magnitude, but not of the phase, with changing field. In contrast, measurement of a non-collinear SDW with the same tip will show a field-dependent phase shift of the stripe pattern of the d*I*/d*V* signal, since the tip gets more sensitive to $M_{{{\text{S}},{ \bot }}}$ as the field increases [9].

#### Revealing noncollinearity of stripe patterns in BLH Fe nanoisland

**Fig. 8:**
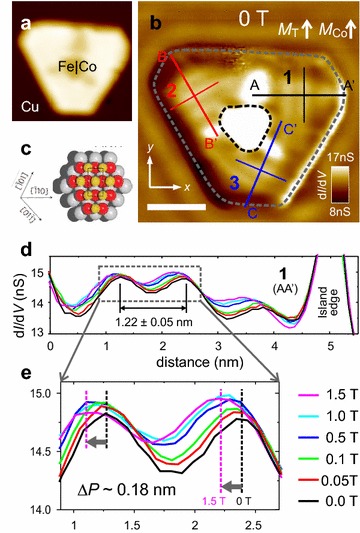
In-field spin-STS of a biatomic-layer-high Fe nanoisland with a Stoner-Wohlfarth tip. **a** CC-STM image of a Fe|Co island on Cu(111). Scale bar is 4 nm long. **b** Hard sphere model of the atomic stacking in a Fe|Co island. The *gray*, *red*, and *yellow spheres* are the Cu, *bottom layer* Fe, and *top layer* Fe, respectively. **c** d*I*/d*V* map of the Fe|Co island shown in **a** measured at 0 T, where both magnetizations of tip ***M***_T_ and Co core ***M***_Co_ point up (*V*_b_ = −0.3 V, *I*_set_ = 3 nA). The island edges and Co core are indicated by the *grey* and *black dashed lines*. The stripe patterns in three (*1*, *2*, *3*) regions originate from the non-collinear cycloidal spin orders in the bridge-stacked bilayer Fe on Cu(111). The *black*, *red* and *blue solid lines* (*arrows*) indicate the directions of the stripes (wave vectors of the cycloidal spin orders), in the regions *1*, *2* and *3*, respectively. **d** Field-dependence of d*I*/d*V* profiles along the *arrow 1* in a, measured with the field sweep of 0–1.5 T. **e** Magnified view of the *grey dashed box* in **d** and is shown for a clarity of field-dependent shifts of the location of the maxima in **d**. The *black* and *purple dotted lines* denote the *peak positions* of the profiles measured at 0 and 1.5 T, respectively (Reprinted from [7] with permission from Nature Publishing Group (Copyright 2014))

Figure 8a, b show CC-STM and d*I*/d*V* images of a Fe|Co island, measured at 0 T. Figure 8c is a hard sphere model representing the atomic stacking in Fe region 1 of b. The Fe regions at the three corners of the island show a stripe contrast along three different directions as indicated by the solid lines superposed along the stripes with the labels 1–3. To resolve the spin ordering in the stripe patterns, we perform in-field spin-STS with the SW tip as characterized by spin-STM on a BLH Co on Cu(111) (Fig. 7). In this tip the direction of ***M***_T_ is tuned by an external field. The magnetic easy axis of the tip is canted by 55 ± 1° from the sample normal. Therefore, it is sensitive to both $M_{{{\text{S}},{ \bot }}}$ *M*_T,||_ and $M_{{{\text{S}},{ \bot }}}$ at 0 T, while it is sensitive only to the $M_{{{\text{S}},{ \bot }}}$ at a field large enough to saturate $M_{{{\text{S}},{ \bot }}}$. Figure 8d shows the field dependence of the d*I*/d*V* profile along the line AA’ perpendicular to the stripe direction of region 1 in Fig. 8b. The wavelength of the stripe pattern, as denoted, is identical with that (1.28 nm) observed in the pure Fe island [7, 20]. Figure 8e shows a zoom-in of the field dependence between two maxima in the profiles, as shown within the grey dashed line in Fig. 8d, for clarity. With increasing magnetic field, the positions of maxima and minima move monotonically from right to left, while the distance between the extrema remains constant. A phase shift of ∆*P* ~ 0.18 nm is measured upon a change of the magnetic field from 0 to 1.5 T. This observation rules out a collinear SDW. Rather, in-field spin-STS identifies a non-collinear-SDW as the spin texture of the stripe contrast.

#### Simulation of phase shift in the field dependence of the stripe pattern

Based on Wortmann et al.’s discussion [15], the d*I*/d*V*|_mag_ (Eq. 7) signal is proportional to the projection of ***M***_S_ to ***M***_T_ at the tip position, i.e., $\hat{\varvec{e}}_{\text{T}} \cdot \hat{\varvec{e}}_{\text{S}}$. Here we calculate a normalized $\varvec{M}_{\text{T}} \cdot \varvec{M}_{\text{S}}$ ($\left. {\varvec{M}_{\text{T}} \cdot \varvec{M}_{\text{S}} } \right|_{\text{norm}}$) with respect to the external field. We model ***M***_T_ as a SW magnet of a magnetic easy axis canted by *α* = 55° from the external field direction, as discussed in Fig. 7c and d. An ab initio study of the sample configuration [7] predicted a Néel-type non-collinearity, where the plane of the spin rotation is parallel with the wave vector of a periodicity *λ*_SH_ = 1.28 nm. Thereby, we call this spin order a “spin-cycloid” in the rest of the paper. Then both the out-of-plane (*M*_S,z_) and in-plane (*M*_S,x_) components of the ***M***_S_ show a sinusoidal position dependence along the *x*-axis. Thus, $\hat{\varvec{e}}_{{\text{S,z}}}$ and $\hat{\varvec{e}}_{{\text{S,x}}}$ can be described by the equations

10 $$\hat{\varvec{e}}_{{\text{S,z}}} = \sin \left( {\frac{2\pi x}{{\lambda_{\text{SH}} }}} \right),\quad\hat{\varvec{e}}_{{\text{S,x}}} = \sin \left( {\frac{2\pi x}{{\lambda_{\text{SH}} }} \mp \frac{\pi }{2}} \right),$$

where the signs ‘−’ and ‘+’ in the formula of $\hat{\varvec{e}}_{{\text{S,x}}}$ indicate a right-rotating (RR) and left- rotating (LR) cycloid, respectively.

**Fig. 9:**
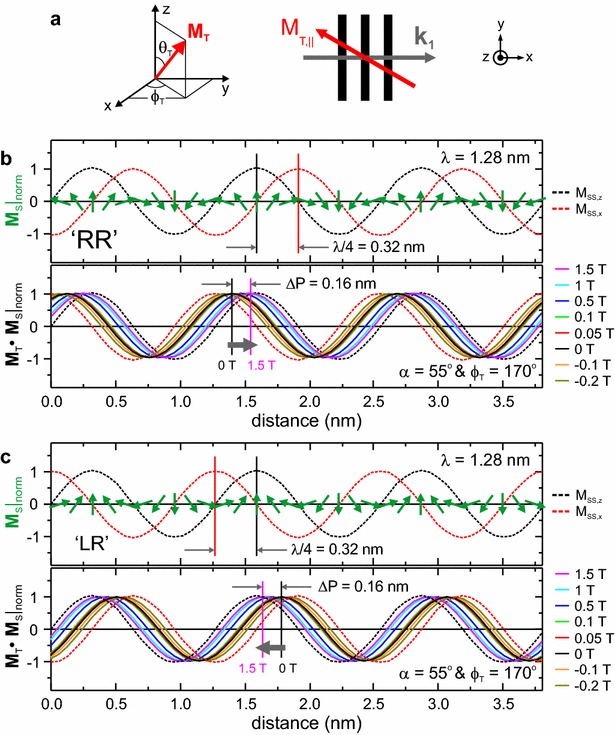
Probing non-collinear cycloidal spin order with a field-tuned tip with Stoner-Wohlfarth magnetization behavior. **a** A description of the tip magnetization ***M***_T_ of a “Stoner-Wohlfarth tip”, with the polar (*θ*_T_) and azimuthal ($\phi$_T_) angles, in the Cartesian coordinate system and a sketch of the geometric relation between the in-plane component of the tip magnetization ***M***_T_, and the wave vector *k*_1_ of the stripe pattern for the region 1 in Fig. 8 **c**. The *black bars* indicate the stripe pattern. **b**, **c** The upper figure of **b** (**c**) shows the *x*-position dependence of out-of-plane and in-plane components of local magnetization of the RR- (LR-) order. The lower figure of **b** (**c**) shows the field dependence of the $\left. {\varvec{M}_{\text{T}} \cdot \varvec{M}_{\text{S}} } \right|_{norm}$ curves as a function of the position *x*, calculated with *θ*_T_ = 55° and *φ*_T_ = 170° for a RR- (LR-) order. The in-plane (*red dotted*) and out-of-plane (*black dotted*) components of the spin cycloidal order are shown in the coordinate system illustrated in **a**. The green arrows in the upper figure of **b** (**c**) indicate the local magnetization variation in space for a RR- (LR-) order. Reprinted from [7] with permission from Nature Publishing Group (Copyright 2014) (Reprinted from [7] with permission from Nature Publishing Group (Copyright 2014))

We calculate the signal induced by the stripe pattern in region 1 in Fig. 8b. Figure 9a shows a description of ***M***_T_, with the polar (*θ*_T_) and azimuthal (*φ*_*T*_) angles, in the Cartesian coordinate system. A sketch of the geometric relation between the in-plane component of the tip magnetization ***M***_T,||_ and the wave vector ***k***_1_ of the stripe pattern for region 1 in Fig. 8b is presented. Figure 9b shows the field dependence $\left. {\varvec{M}_{\text{T}} \cdot \varvec{M}_{\text{S}} } \right|_{\text{norm}}$ as a function of position *x* (lower figure), calculated with *α* = 55° and *φ*_T_ = 170° for a RR-cycloid, with the *x*-position dependent out-of-plane (black dotted) and in-plane (red dotted) components of ***M***_S_(*x*) (upper figure). Figure 9c shows the results of the corresponding calculation but for a LR-cycloid. Note the left-to-right (right-to-left) shift of the maxima of $\left. {\varvec{M}_{\text{T}} \cdot \varvec{M}_{\text{S}} } \right|_{\text{norm}}$ from 0 to 1.5 T for the RR- (LR-) cycloid for the given azimuthal angle of ***M***_T_. For the stripe contrast in region 1 of the island in Fig. 8, we observe that the extrema of the d*I*/d*V* curves shift from right to left. Interestingly, the three cases show different amounts of phase shifts with increasing field (Supplementary Information of [7]). We perform a quantitative analysis of the phase shift and its dependence on stripe orientation and field. An excellent agreement between the experiments and simulations reveals that the field dependence of the phase shifts is determined by a given orientation of the tip magnetization.

### Spin-STM of a non-collinear magnetic state with a superparamagnetic tip

**Fig. 10:**
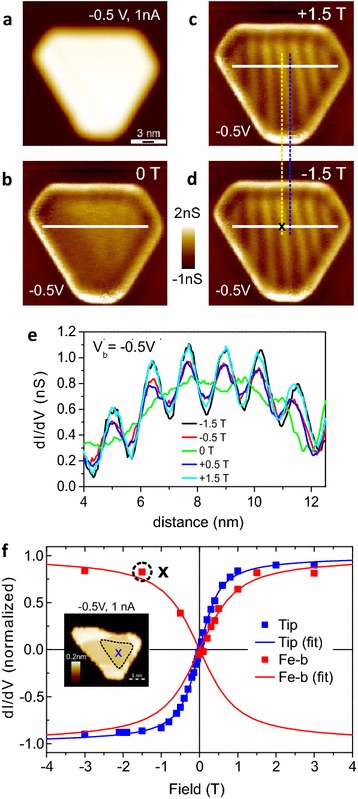
Spin-STM of thermally fluctuating magnetic order with a field-tuned superparamagnetic tip.** a**–**d** CC-STM (**a**) and d*I*/d*V* maps at 0 T (**b**), +1.5 T (**c**), and −1.5 T (**d**) of a bilayer bridge-stacked Fe nanoisland measured with a superparamagnetic Fe-coated W tip at 10 K. (*V*_b_ = − 0.5 V, *I*_set_ = 1 nA). **e** dI/dV profiles along the line depicted in the CC-STM image in **a**, measured at −1.5 T (*black*), −0.5 T (*red*), 0 (*green*), +0.5 T (*blue*), +1.5 T (*cyan*). **f** Field dependence of d*I*/d*V* contrast of the stripe pattern (*red*) measured at a bright center as marked by the cross in **d**. Field dependence of the d*I*/d*V* contrast measured at the center of the Co (*blue*) of the Fe|Co island as indicated in the *inset*, with the same tip as used for measuring the Fe island. The *solid curves* are fits of the data to the Langevin equation, for the Fe (*red*) and Fe|Co (*blue*) nanoislands. All the data are normalized with the saturation values obtained from the Langevin fits

Figure 10a is CC-STM image of a BLH Fe island on Cu(111) in bridge-site stacking of the topmost atoms, measured with a Fe-coated W tip. Figures 10b–d are d*I*/d*V* images of the same island as that of Fig. 10a measured at 0 T, +1.5 T, and −1.5 T, respectively. The stripe patterns in Fig. 10c, d indicate a cycloidal spin order as discussed in the text above describing Figs. 8 and 9. However, no stripe contrast is observed in the d*I*/d*V* map at zero field. We obtained d*I*/d*V* images under an external field from −3 to +3 T along the sample normal. We show in Fig. 10e the stripe contrast at 5 field values, measured along a direction perpendicular to the stripe patterns as indicated by the white lines in Fig. 10b–d. Figure 10f shows the field dependence of the stripe contrast (red), peak-to-peak amplitude of the contrast oscillation at each field followed by a normalization with the saturation value from the Langevin fitting (Eq. 8). The inspection of Fig. 10e reveals two aspects: (1) The contrast at a given position increases for increasing applied field. No stripe contrast is observed at zero field. The stripe contrast saturates at ~±1.5 T. (2) The contrast depends only on the magnitude of the field, but not on the sign of the field. The extrema positions of the stripe patterns as indicated by the yellow and blue dashed lines in Fig. 10c and d remain unchanged. To obtain a quantitative insight into the field dependence of the stripe contrast (red curve in Fig. 10f), we also obtained the field dependence of d*I*/d*V* signals measured at the center of the Co core of a Fe|Co island (inset) with the same tip. This measurement on the Co reference sample definitely reflects the response of the tip to the applied field (blue in Fig. 10f). This tip behaves as a superparamagnetic particle. The larger slope near zero field for measurements on Co (blue curve) as compared to measurements on Fe (red curve), clearly implies a sizable contribution of the response of the sample magnetic order to the measured quantity of the d*I*/d*V* signal (red). In addition, the results imply a ‘non-hysteretic’ and ‘monotonic’ response of the Fe magnetic order to the applied field.

The field dependence of the magnetization ***M***_T_ of a superparamagnetic tip can be written as ***M***_T_(*H*) = −***M***_T_(−*H*) (blue curve in Fig. 10f). The field dependence of the d*I*/d*V* signal of the pure Fe island shown in Fig. 10 reveals a relation of d*I*/d*V* (−*H*) = d*I*/d*V* (*H*) (red). Then, the relation given by Wortmann et al., d*I*/d*V*|_mag_ = ***m***_T_ · ***m***_Fe_, resolves the influence of the sign reversal in *H* on the magnetization density of state ***m***_Fe_ (see Sect. 1.2) through a simple calculation

11 $$\left. {\frac{dI}{dV}\left( { - H} \right)} \right|_{\text{mag}} = \varvec{m}_{\text{T}} \left( { - H} \right) \cdot \varvec{m}_{\text{Fe}} \left( { - H} \right) \Leftrightarrow \left. {\frac{dI}{dV}\left( H \right)} \right|_{\text{mag}} = \varvec{m}_{\text{T}} \left( H \right) \cdot \varvec{m}_{\text{Fe}} \left( H \right),$$

which leads to a relation ***m***_S_(−*H*_ext_) = −***m***_S_(*H*_ext_). This, concurrent with the above-mentioned ‘non-hysteretic’ and ‘monotonic’ field dependence, provides an important conclusion on the magnetic property of the non-collinear spin order in the Fe island. The stripe order in Fe island responds to the external field as an effective magnetic moment, which is thermally fluctuating at a given measurement temperature. We show in the next section the significance of this aspect to obtain a spin-polarization map of the Fe stripe phase from the data set shown in Fig. 10a–c.

### Spin-polarization of magnetically ordered nanostructures

#### Differential conductance asymmetry

The magnetic configuration of a sample is determined by its spin-dependent electronic structure, giving rise to spin polarization of the electronic density of states. To investigate the spin polarization of a sample with spin-STM, the asymmetry of the differential conductance, *A*_d*I*/d*V*_, is introduced. The asymmetry is defined as [5]

12 $$A_{{{\text{dI}}/{\text{dV}}}} = \frac{{\left. {{\text{d}}I/{\text{d}}V} \right|_{\text{AP}} - \left. {{\text{d}}I/{\text{d}}V} \right|_{\text{P}} }}{{\left. {{\text{d}}I/{\text{d}}V} \right|_{\text{AP}} + \left. {{\text{d}}I/{\text{d}}V} \right|_{\text{P}} }}$$

**Fig. 11:**
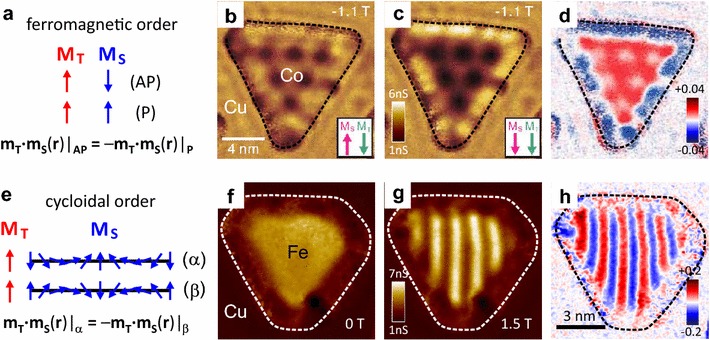
Differential conductance asymmetry *A*_dI/dV_. **a** Two magnetic configurations in spin-STM measurements, *AP* and *P*, corresponding to two distinct magnetic states of a bilayer Co nanoisland, pointing up and down, respectively. **b**, **c** d*I*/d*V* images of the Co nanoisland ‘A’ in Fig. 5 **a** measured at μ_0_ *H*_ext_ = −1 T and *V*_b_ = + 0.03 V for *AP* (**b**) and *P* (**c**) states. **d** *A*_dI/dV_ map calculated from the d*I*/d*V* images of **b** and **c**. **e** Two relative magnetization configurations of spin-STM measurements, corresponding to two distinct magnetic states of a bilayer Fe nanoisland, α and β. **f**, **g** d*I*/d*V* images of a Fe nanoisland, measured at external fields of (**b**) 0 T and (**c**) a value ≥ *H*_sat_. **h** *A*_dI/dV_ map calculated from the d*I*/d*V* images of **f** and **g**. **b**–**d** Reprinted from [18] with permission from Institute of Physics (Copyright 2014). **f**–**h** Reprinted from [20] with permission from Nature Publishing Group (Copyright 2016)

In case of a ferromagnetic sample of bistable magnetization, *A*_dI/dV_ is calculated from the d*I*/d*V* signals recorded under *P* and *AP* to the unit vector of magnetization configurations, i.e. ***ê***_T_·***ê***_S_ = ±1, as schematically illustrated in Fig. 11a. As introduced in Sect. 1.2, Wortmann et al. derived the description of the d*I*/d*V* signal measured by spin-STM [15] (Eq. 7). Substitution of their result into the Eq. 12 leads to

13 $$A_{{{\text{dI}}/{\text{dV}}}} = - P_{\text{T}} P_{\text{S}} ,$$

which links the d*I*/d*V* asymmetry, *A*_dI/dV_, to the spin polarization of the sample at the tip apex position, *P*_S_(***R***_T_).

Equations 11 and 12 were applied to the Co island ‘A’ in Fig. 5a to extract the spatial distribution of its spin-polarization. Figure 11a, b are two d*I*/d*V* images on the island measured at μ_0_ *H* = −1.1 T, with (b) *AP* and (c) *P* magnetization configurations, as indicated in Fig. 5c. Figure 11d is the *A*_dI/dV_ map, at the given bias voltage *V*_b_, calculated from the d*I*/d*V* images in Fig. 11b and c. Oka et al. extracted a set of energy-resolved *A*_dI/dV_ maps of this Co island, which led to the disclosure of the electronic nature of “spin-dependent quantum interference within a single nanostructure” [5].

#### Differential conductance asymmetry of non-collinear magnetic order

In case of a helical (or cycloidal) spin order, the local magnetization rotates with a spatial period. Thus, the spatially averaged magnetization is zero. As discussed in Sect. 4.2, a sign reversal of the external field induces a corresponding reversal in that of the local magnetization, ***M***_S_(***r***; −*H*) = −***M***_S_(***r***; *H*), indicative of two distinct antiparallel magnetic states at each position ***r***. In addition, the power of spin-STM, which allows to resolve the local magnetic signal down to the atomic scale, makes a study on the local spin-polarization of the cycloidal order in the bilayer Fe island feasible. Although the definition of *AP* and *P* configurations is not applicable to this case due to the periodic change of the local magnetization, one can clearly distinguish two distinct magnetic states, as sketched in Fig. 11e. Combined with the tip magnetic state, we introduce two magnetic configurations *α* and *β*, hence ***M***_S,_(***r***) = −***M***_S,*β*_(***r***), analogous to the *AP* and *P* configurations in the case of Fig. 11a. The asymmetry *A*_d*I*/d*V*_ for the cycloidal spin order in the Fe island is defined as

14 $$A_{{{\text{dI}}/{\text{dV}}}} = \frac{{\left. {{\text{d}}I/{\text{d}}V} \right|_{\alpha } - \left. {{\text{d}}I/{\text{d}}V} \right|_{\beta } }}{{\left. {{\text{d}}I/{\text{d}}V} \right|_{\alpha } + \left. {{\text{d}}I/{\text{d}}V} \right|_{\beta } }}$$

With substitution of the Eqs. 7, 14 becomes

15 $$A_{{{\text{dI}}/{\text{dV}}}} = \frac{{n_{\text{T}} n_{\text{S}} + \varvec{m}_{\text{T}} \cdot \varvec{m}_{{{\text{S}},\alpha }} - n_{\text{T}} n_{\text{S}} - \varvec{m}_{\text{T}} \cdot \varvec{m}_{{{\text{S}},\beta }} }}{{n_{\text{T}} n_{\text{S}} + \varvec{m}_{\text{T}} \cdot \varvec{m}_{{{\text{S}},\alpha }} + n_{\text{T}} n_{\text{S}} + \varvec{m}_{\text{T}} \cdot \varvec{m}_{{{\text{S}},\beta }} }} = - \frac{{\varvec{m}_{\text{T}} \cdot \varvec{m}_{{{\text{S}},\alpha }} }}{{n_{\text{T}} n_{\text{S}} }}$$

This leads to a link between the symmetry *A*_dI/dV_ and the spin polarization of the sample at the tip apex position, *P*_S_(***R***_T_) in the form

16 $$A_{{{\text{dI}}/{\text{dV}}}} = - P_{\text{T}} P_{\text{S}} \left( {\varvec{R}_{\text{T}} } \right)\cos \theta ,$$

where *θ* is the angle between ***M***_T_ and ***M***_S_ (see Fig. 1a). The asymmetry *A*_dI/dV_ corresponds to the projection of *P*_S_ onto the tip magnetization direction.

If a superparamagnetic tip (Fig. 6) is used in spin-STM/S of the cycloidal spin order in the bilayer Fe island, one is not able to have the above-mentioned two magnetization configurations (*α* and *β*) because the tip magnetization will also be reversed by the sign reversal of the applied field, as discussed in Fig. 10. This always results in the numerator of the Eq. (14) to be zero. We introduce a procedure to overcome this obstacle in the following discussion. A careful inspection of the right hand side of Eq. 15 indicates that the denominator and numerator are no other than non-magnetic and magnetic terms of the d*I*/d*V* signals for the configuration *α*, respectively. These two contributions are provided by the d*I*/d*V*|_*H*=0_ and d*I*/d*V*|_*α*_ – d*I*/d*V*|_*H*=0_ data, respectively. Figure 11f, g show the d*I*/d*V*|_*H*=0_ and d*I*/d*V*|_*α*_ maps of a bilayer Fe nanoisland measured with a superparamagnetic tip, and Fig. 11h is its *A*_d*I*/d*V*_ map, derived from Fig. 11f and g as given by Eq. 15. Fischer et al. extracted a set of energy-resolved *A*_dI/dV_ maps of this Fe island, which were successfully utilized to reveal the “spinor nature of electronic states in nanosize non-collinear magnets” [20].

## Concluding remarks

In the recent two decades, spin-STM has evolved into a reliable and versatile tool for spatial mapping of local magnetic structures of collinear and non-collinear spin textures on the nano-scale. The unsurpassed lateral resolution turned spin-STM into a unique tool to collect the magnetic information down to the atomic scale of single nanostructures. The tunneling nature of STM/S inevitably couples contributions from both tip and sample in the measured signals, *I*(*V*) and d*I*/d*V*(*V*). Understanding the tip contribution has been a challenging issue for a reliable analysis of the sample properties. Undoubtedly, this is also true in the spin-STM/S measurements. In this review, we introduced some examples of tip characterization in spin-STM/S experiments. This analysis has advanced the quantitative physical understanding of spin textures in nanostructures.

Based on in situ reference measurements on the bistable out-of-plane magnetization of biatomic-layer-high Co nanoislands on Cu (111), we characterized magnetic states of spin-STM tips quantitatively. Temperature-dependent in-field spin-STM/S gives a quantitative characterization of tips in the superparamagnetic state. The analysis reveals stray field at the tip position induced by the sample magnetization. In-field spin-STM/S combined with the Stoner-Wohlfarth (SW) model [55] for tip magnetization elucidates the orientation of the magnetic anisotropy of bistable ferromagnetic tips. Our studies show that the SW model within the framework of the superparamagnetic criterion indeed explains the magnetic response of tips of various types, which give rise to distinctly different field-dependence of the spin-STS signal.

In-field STM/S in combination, such sophisticated tip characterization allows to characterize the local magnetic order and local spin-dependent electronic structure of magnetic nanostructures. Field-tuning of the orientation of a magnetically bistable tip was applied to reveal the non-collinearity of the one-dimensional periodic magnetic order in Fe nanoislands. The spin-dependent d*I*/d*V* mapping of Co and Fe nanostructures, in antiparallel and parallel configurations of tip and sample magnetizations, provides differential conductance asymmetry maps, which directly link spin-STS data to the local spin-polarization within a single nanostructure.

In spite of the remarkable advances to date in the characterization of the tip states by exploiting external field control, the spin-STM research field still requires more effort to overcome the following existing barriers. It is a goal to define the tip apex to maintain a specific structural configuration on the atomic scale during a set of measurements over periods ranging from days to months. However, a specific recipe of macroscopic (or ex situ) tip preparation does not seem to play a decisive role for the resulting magnetic behavior, as we demonstrated in this review (Fig. 4). Up to now, preparation and tuning of a tip is extremely tricky. Key ingredients are in situ treatments by voltage pulses. The tip never comes back to its original form once the tip loses its microscopic configuration by crashes with the sample, which is unfortunately common in STM measurement. Manipulation technique of single atoms on a surface using STM tips [10, 58–61] has been advanced significantly through precise estimation of the force required to move an individual atom on a surface [62, 63]. This allows to reliably attach/detach individual atoms to/from the tip apex without any change of the rest of the tip. In addition, complete control of the vector nature of the tip and sample magnetizations call for a “vector magnet field”, which comes with a high price tag. Future advance in spin-STM is guaranteed by the combination of these advanced techniques.

## References

[1] Bode M (2003). Spin-polarized scanning tunnelling microscopy. Rep. Prog. Phys..

[2] Wulfhekel W, Kirschner J (2007). Spin-polarized scanning tunneling microscopy of magnetic structures and antiferromagnetic thin films. Annu. Rev. Mater. Res..

[3] Wiesendanger R (2009). Spin mapping at the nanoscale and atomic scale. Rev. Mod. Phys..

[4] Oka H, Brovko OO, Corbetta M, Stepanyuk VS, Sander D, Kirschner J (2014). Spin-polarized quantum confinement in nanostructures: scanning tunneling microscopy. Rev. Mod. Phys..

[5] Oka H, Ignatiev PA, Wedekind S, Rodary G, Niebergall L, Stepanyuk VS, Sander D, Kirschner J (2010). Spin-dependent quantum interference within a single magnetic nanostructure. Science.

[6] Ouazi S, Wedekind S, Rodary G, Oka H, Sander D, Kirschner J (2012). Magnetization reversal of individual co nanoislands. Phys. Rev. Lett..

[7] Phark SH, Fischer JA, Corbetta M, Sander D, Nakamura K, Kirschner J (2014). Reduced-dimensionality-induced helimagnetism in iron nanoislands. Nat. Commun..

[8] Heinze S, Bode M, Kubetzka A, Pietzsch O, Nie X, Blügel S, Wiesendanger R (2000). Real-space imaging of two-dimensional antiferromagnetism on the atomic scale. Science.

[9] Bode M (2007). Chiral magnetic order at surfaces driven by inversion asymmetry. Nature.

[10] Loth S, Baumann S, Lutz CP, Eigler DM, Heinrich AJ (2012). Bistability in atomic-scale antiferromagnets. Science.

[11] Tedrow PM, Meservey R (1973). Spin polarization of electrons tunneling from films of Fe Co, Ni, and Gd. Phys. Rev. B.

[12] Meservey R, Tedrow PM, Fulde P (1970). Magnetic field splitting of the quasiparticle states in superconducting aluminum films. Phys. Rev. Lett..

[13] Julliere M (1975). Tunneling between ferromagnetic films. Phys. Lett..

[14] Kittel C (1949). Physical theory of ferromagnetic domains. Rev. Mod. Phys..

[15] Wortmann D, Heinze S, Kurz P, Bihlmayer G, Blügel S (2001). Resolving complex atomic-scale spin structures by spin-polarized scanning tunneling microscopy. Phys. Rev. Lett..

[16] Bardeen J (1961). Tunneling from a many-particle point of view. Phys. Rev. Lett..

[17] Tersoff J, Hamann DR (1985). Theory of the scanning tunneling microscope. Phys. Rev. B.

[18] Sander D, Phark SH, Corbetta M, Fischer JA, Oka H, Kirschner J (2014). The impact of structural relaxation on spin polarization and magnetization reversal of individual nano structures studied by spin-polarized scanning tunneling microscopy. J. Phys.: Condens. Matter.

[19] Phark SH, Fischer JA, Corbetta M, Sander D, Kirschner J (2013). Superparamagnetic response of Fe-coated W tips in spin-polarized scanning tunneling microscopy. Appl. Phys. Lett..

[20] Fischer JA, Sandratskii LM, Phark SH, Ouazi S, Pasa AA, Sander D, Parkin SSP (2016). Probing the spinor nature of electronic states in nanosize non-collinear magnets. Nat. Commun..

[21] Crommie MF, Lutz CP, Eigler DM (1993). Imaging standing waves in a two-dimensional electron gas. Nature (London).

[22] Diekhöner L, Schneider MA, Baranov AN, Stepanyuk VS, Bruno P, Kern K (2003). Surface states of cobalt nanoislands on Cu(111). Phys. Rev. Lett..

[23] Biedermann A, Rupp W, Schmid M, Varga P (2006). Coexistence of fcc- and bcc-like crystal structures in ultrathin Fe films grown on Cu(111). Phys. Rev. B.

[24] Gerhard L (2010). Magnetoelectric coupling at metal surfaces. Nat Nanotech..

[25] Rodary G, Wedekind S, Oka H, Sander D, Kirschner J (2009). Characterization of tips for spin-polarized scanning tunneling microscopy. Appl. Phys. Lett..

[26] Corbetta M, Ouazi S, Borme J, Nahas Y, Donati F, Oka H, Wedekind S, Sander D, Kirschner J (2012). Magnetic response and spin polarization of bulk Cr tips for in-field spin-polarized scanning tunneling microscopy. Jpn. J. Appl. Phys..

[27] Maekawa S, Gäfvert U (1982). Electron tunneling between ferromagnetic films. IEEE Trans. Magn..

[28] Miyazaki T, Tezuka N (1995). Giant magnetic tunneling effect in Fe/Al2O3/Fe junction. J. Magn. Magn. Mater..

[29] Moodera JS, Kinder LR, Wong TM, Meservey R (1995). Large magnetoresistance at room temperature in ferromagnetic thin film tunnel junctions. Phys. Rev. Lett..

[30] Prins MWJ, Abraham DL, van Kempen H (1993). Spin-dependent transmission at ferromagnet/semiconductor interfaces. J. Magn. Magn. Mater..

[31] Tedrow PM, Meservey R (1971). Direct observation of spin-state mixing in superconductors. Phys. Rev. Lett..

[32] Tedrow PM, Meservey R (1971). Spin-dependent tunneling into ferromagnetic nickel. Phys. Rev. Lett..

[33] Wulfhekel W, Kirschner J (1999). Spin-polarized scanning tunneling microscopy on ferromagnets. Appl. Phys. Lett..

[34] Gao CL, Schlickum U, Wulfhekel W, Kirschner J (2007). Mapping the surface spin structure of large unit cells: reconstructed Mn films on Fe(001). Phys. Rev. Lett..

[35] von Bergmann K, Menzel M, Serrate D, Yoshida Y, Schröder S, Ferriani P, Kubetzka A, Wiesendanger R, Heinze S (2012). Tunneling anisotropic magnetoresistance on the atomic scale. Phys. Rev. B.

[36] Néel N, Schröder S, Ruppelt N, Ferriani P, Kröger J, Berndt R, Heinze S (2013). Tunneling tunneling anisotropic magnetoresistance on the atomic scale. Phys. Rev. Lett..

[37] Caffrey NM, Schröder S, Ferriani S Heinze (2014). Tunneling anisotropic magnetoresistance effect of single adatoms on a noncollinear magnetic surface. J. Phys.: Condens. Matter.

[38] Cavallini M, Biscarini F (2000). Electrochemically etched nickel tips for spin polarized scanning tunneling microscopy. Rev. Sci. Instr..

[39] Bassi AL, Casari CS, Cattaneo D, Donati F, Foglio S, Passoni M, Bottani CE, Biagioni P, Brambilla A, Finazzi M, Ciccacci F, Duò L (2007). Appl. Phys. Lett..

[40] Minakov AA, Shvets IV (1990). On the possibility of resolving quantization axes of surface spins by means of a scanning tunneling microscope with a magnetic tip. Surf. Sci..

[41] Murphy S, Osing J, Shvets IV (1999). Fabrication of submicron-scale manganese-nickel tips for spin-polarized STM studies. Appl. Surf. Sci..

[42] Mariotto G, Murphy S, Shvets IV (2002). Charge ordering on the surface of Fe3O4(001). Phys. Rev. B.

[43] Ceballos SF, Mariotto G, Murphy S, Shvets IV (2003). Fabrication of magnetic STM probes and their application to studies of the Fe3O4(001) surface. Surf. Sci..

[44] Feuchtwang TE, Cutler PH, Schmit J (1978). Surf. Sci..

[45] Nagai S, Hata K, Oka H, Sander D, Kirschner J (2014). Atomic structure and spin polarization at the apex of tips used in spin-polarized scanning tunneling microscopy. Appl. Phys. Exp..

[46] Hashizume T, Hasegawa Y, Kamiya I, Ide T, Sumita I, Hyodo S, Sakurai T, Tochihara H, Kubota M, Murata Y (1990). J. Vac. Sci. Technol., A.

[47] Pietzsch O, Kubetzka A, Bode M, Wiesendanger R (2004). Spin-polarized scanning tunneling spectroscopy of nanoscale cobalt islands on Cu(111). Phys. Rev. Lett..

[48] Yayon Y, Brar VW, Senapati L, Erwin SC, Crommie MF (2007). Observing spin polarization of individual magnetic adatoms. Phys. Rev. Lett..

[49] Iacovita C, Rastei MV, Heinrich BW, Brumme T, Kortus J, Limot L, Bucher JP (2008). Visualizing the spin of individual cobalt-phthalocyanine molecules. Phys. Rev. Lett..

[50] de la Figuera J, Prieto JE, Ocal C, Miranda R (1993). Scanning-tunneling-microscopy study of the growth of cobalt on Cu(111). Phys. Rev. B.

[51] Negulyaev NN, Stepanyuk VS, Bruno P, Diekhöner L, Wahl P, Kern K (2008). Bilayer growth of nanoscale Co islands on Cu(111). Phys. Rev. B.

[52] Bean CP, Livingston JD (1959). Superparamagnetism. J. Appl. Phys..

[53] Billas IML, Becker JA, Chârelain A, de Heer WA (1993). Magnetic moments of iron clusters with 25 to 700 atoms and their dependence on temperature. Phys. Rev. Lett..

[54] Stearns MB (1986). Magnetic properties of 3d, 4d, and 5d elements alloys and compounds (Landolt-Bornstein numerical data and functional relationships in science and technology group III).

[55] Stoner E, Wohlfarth E (1948). A mechanism of magnetic hysteresis in heterogeneous alloys. Philos Trans R Soc A Phys Mech Eng Sci.

[56] Overhauser AW (1962). Spin density waves in an electron gas. Phys. Rev..

[57] Fawcett E (1988). Spin-density-wave antiferromagnetism in chromium. Rev. Mod. Phys..

[58] Crommie MF, Lutz CP, Eigler DM (1993). Confinement of electrons to quantum corrals on a metal surface. Science.

[59] Manoharan HC, Lutz CP, Eigler DM (2000). Quantum mirages formed by coherent projection of electronic structure. Nature.

[60] Heinrich AJ, Lutz CP, Gupta JA, Eigler DM (2002). Molecule cascades. Science.

[61] Hirjibehedin CF, Lutz CP, Heinrich AJ (2006). Spin coupling in engineered atomic structures. Science.

[62] Ternes M, Lutz CP, Hirjibehedin CF, Giessibl FJ, Heinrich AJ (2008). The force needed to move an atom on a surface. Science.

[63] Ternes M, González C, Lutz CP, Hapala P, Giessibl FJ, Jelínek P, Heinrich AJ (2011). Interplay of conductance, force, and structural change in metallic point contacts. Phys. Rev. Lett..

[64] Chikazumi S (1997). Physics of ferromagnetism, international series of monographs on physics.

